# How is the use of research evidence in health policy perceived? A comparison between the reporting of researchers and policy-makers

**DOI:** 10.1186/s12961-018-0345-6

**Published:** 2018-07-20

**Authors:** Moriah E. Ellen, John N. Lavis, Einav Horowitz, Robin Berglas

**Affiliations:** 1 Department of Health Systems Management, Guilford Glazer Faculty of Business and Management and Faculty of Health Sciences, Ben-Gurion University of the Negev, P.O. Box 653, 84105 Beer-Sheva, Israel; 2 McMaster Health Forum, McMaster University, 1280 Main Street West, Hamilton, ON L8S-4L6 Canada; 3 Institute for Health Policy, Management and Evaluation, University of Toronto, 4th Floor, 155 College St, Toronto, ON M5T 3M6 Canada; 4 Centre for Health Economics and Policy Analysis, McMaster University, 1280 Main Street West, Hamilton, ON L8S-4L6 Canada; 5 Department of Political Science, McMaster University, 1280 Main Street West, Hamilton, ON L8S-4L6 Canada; 6 Department of Global Health and Population, Harvard School of Public Health, 677 Huntington Ave, Boston, MA 02115 United States of America; 7 Israeli Center for Technology Assessment in Health Care, Gertner Institute, Chaim Sheba Medical Center, 52621 Tel Hashomer, Israel; 8 Independent Researcher, Jerusalem, Israel

**Keywords:** Knowledge transfer and exchange, Policy-maker, Researcher, Evidence-informed policy-making

## Abstract

**Background:**

The use of health policy and systems research (HPSR) to inform health policy-making is an international challenge. Incorporating HPSR into decision-making primarily involves two groups, namely researchers (knowledge producers) and policy-makers (knowledge users). The purpose of this study was to compare the perceptions of Israeli health systems and policy researchers and health services policy-makers regarding the role of HPSR, factors influencing its uses and potential facilitators and barriers to HPSR, and implementation of knowledge transfer and exchange (KTE) activities.

**Methods:**

A cross-sectional survey was administered to researchers and policy-makers in Israel. The survey consisted of seven closed questions. Descriptive analyses were carried out for closed-ended questions and comparative analysis were conducted between groups using the χ^2^ test.

**Results:**

A total of 37 researchers and 32 policy-makers responded to the survey. While some views were in alignment, others showed differences. More policy-makers than researchers perceived that the use of HPSR in policy was hindered by practical implementation constraints, whereas more researchers felt that its use was hindered by a lack of coordination between knowledge producers and users. A larger percentage of policy-makers, as compared to researchers, reported that facilitators to the KTE process are in place and a larger percentage of researchers perceived barriers within the KTE environment. A larger percentage of policy-makers perceived KTE activities were in place as compared to researchers. Results also showed large differences in the perceptions of the two groups regarding policy formulation and which organisations they perceived as exerting strong influence on policy-making.

**Conclusions:**

This research demonstrated that there are differences in the perceptions of knowledge producers and users about the process of KTE. Future work should focus on minimising the challenges highlighted here and implementing new KTE activities. These activities could include making the researchers aware of the most effective manner in which to package their results, providing training to policy-makers and assuring that policy-makers have technical access to appropriate databases to search for HPSR. These results underscore the need for the groups to communicate and clarify to each other what they can offer and what they require.

**Electronic supplementary material:**

The online version of this article (10.1186/s12961-018-0345-6) contains supplementary material, which is available to authorized users.

## Background

The process of health policy decision-making is complex. There is international awareness about the need to bridge the gap between research, practice and policy [1], as the decision-making process varies between settings and is not viewed uniformly by different key players [2]. One factor influencing the decision-making process is health policy and systems research (HPSR). Incorporating HPSR into decision-making involves two groups, namely researchers, who produce knowledge, and health policy-makers, who use the created knowledge in their attempt to formulate effective and efficient health policies. Previous studies identified possible reasons why evidence is not being used to inform policy [1–6]. Some of those findings stem from the political and economic constraints over which health policy researchers and policy-makers have little influence. Yet, there are areas which have been identified as contributing to the evidence-to-policy gap over which researchers and policy-makers can effect change. Despite the efforts expended by researchers, if research from knowledge producers is not considered by policy-makers to be relevant, packaged in ways in which it can be applied to policy formulation, or readily accessible when decision-makers need it, then the likelihood of decision-makers utilising the research evidence is low [2, 7–10]. Policy-makers need to be receptive to evidence-informed policy-making. On a practical level, this means that they must be equipped with the knowledge and ability to access the information and apply the research evidence. If research uptake by policy-makers is not optimal because infrastructures for obtaining research findings in a timely manner are not effectively employed, then it is unlikely that policy will be informed by the best available evidence [2].

Bridging the gap between knowledge producers and knowledge users is an international challenge [11–16]. The process of knowledge transfer and exchange (KTE) is one approach that has been employed to bridge the ‘know-do’ gap [17]. KTE is defined as “*a dynamic and iterative process that includes synthesis, dissemination, exchange and ethically sound application of knowledge to improve the health of* [citizens], *provide more effective health services and products and strengthen the health care system*” [18]. Simply providing evidence through publications or meetings is an insufficient means to get research evidence into policy decisions [19]. In a time where transdisciplinary research encourages collaboration and creative problem solving, approaches to support evidence-informed decision-making are becoming more adapted and utilised in the healthcare context, and different theories and frameworks are being presented to aid in the dissemination and implementation of research knowledge [20]. KTE is particularly vital in healthcare because knowledge can be fragmented, research proliferation is immense, and the cost of bad decision-making or slow knowledge implementation in healthcare can have dire consequences [21, 22].

Numerous frameworks have been developed, describing an array of potential initiatives related to evidence-informed policy- and decision-making [20, 23–30]. The framework developed by Lavis et al., and modified by others [14, 25, 31, 32], assists in assessing country level efforts related to KTE and provides insight as to which elements a health system should have in place. The first element focuses on the local climate and context in order to assess feasibility and possible implementation challenges. The second element, linkage and exchange, is the building block for the remaining efforts and focuses on creating strong links between knowledge producers and users to enhance the transfer of research into practice. Knowledge creation is the next element, which must be timely and applicable for knowledge users. ‘Push’ and ‘pull’ efforts are also important, and respectively push knowledge out to necessary groups or pull it into those groups from within. Facilitating pull efforts is the next element which focuses on making knowledge accessible for policy-makers and facilitating the identification and use of relevant research. The final element is evaluating the various aforementioned activities to inform future efforts. Understanding how these elements inform the functioning of health systems and those involved in the policy-making process can be the key to understanding what can be changed to make these systems more effective and efficient.

Understanding the main actors’ views about the process can assist in identifying the barriers and facilitators for KTE, minimising barriers and building upon the facilitators. Globally, there have been several qualitative studies comparing the perceptions of researchers and policy-makers, exploring their areas of agreement and disagreement. It is valuable to gain insights from different health systems as solutions are dynamic; certain factors may be applicable across contexts, while others may only have local application. Campbell et al. [3] interviewed researchers separately from policy-makers and reported perceptions from each group regarding getting research into policy. Respondents reported their perceptions of research being used to inform policy, if policy-makers found research accessible and useful, communication and exchange between researchers and policy, and their suggestions of how to increase the use of research in policy. Each group noted that, although research evidence was used in policy planning, more could be done to increase its use in policy. Petticrew et al. [33] and Whitehead et al. [34] conducted two studies looking at the evidence base for policies to reduce health inequalities and examined researcher and policy-maker views on the use of evidence in policy-making. They reported a significant congruence between researchers and policy-makers, suggesting that there may be a common understanding between them and that both groups acknowledged the need to promote an evaluative culture including training researchers to go beyond describing the research towards evaluating the outcomes of interventions. The studies underscored the benefits of the two parties working together, as indicated in other studies [35–37]. The use of health systems evidence by policy-makers in Eastern Mediterranean countries was studied by El Jardali et al. [38] and, although they described differences by region, they did not compare knowledge producer and knowledge user perceptions. Work has also been performed in lower and middle-income countries. For example, Tomson et al. [39] and Jonson et al. [40] examined research evidence usefulness in policy implementation from the perspectives of health services researchers and health policy decision-makers in Laos. Many of the health services researchers in their study were policy-makers who were taught research skills as part of the study. These policy-based researchers facilitated “*getting research into policy and practice*” [39]. Ultimately, regardless of the specific results in each study, all underscored the benefits of researchers and policy-makers working together.

The Israeli healthcare system is held in high regard across the world, providing high quality health services with good outcomes while maintaining manageable levels of expenditures [41]. Like many countries, Israel faces challenges such as scarce resources and the need to make rapid policy decisions. However, while Israel has just as many national policy issues as much larger countries, it has fewer researchers to study them. Although all countries supplement their own research with external sources for formulating policy (e.g. conducting systematic reviews), Israel’s more limited capacity to devote time, effort and money to research may make prioritising what research should be done in-house and when to rely more on outside research much more significant. This can impact the interaction between researchers and policy-makers. On the other hand, the smaller size means that, in many cases, researchers and policy-makers know each other on a personal level, which can influence the nature and tone of their relationship.

The Israeli health system is comprised of the Ministry of Health (MOH), which has overall responsibility for the health of the population and functioning of the health system, four health insurance funds and other non-profit organisations. Many of the decisions, including policy-making, are made at the national level (e.g. coverage under the National Health Insurance Law, nationwide public health initiatives, emergency preparedness, allocation of hospital beds and special devices), but each of the health insurance funds can also make their own internal policy decisions.

In Israel, the high-level policy-makers (e.g. the Minister of Health) are replaced frequently and may possess limited knowledge or experience with certain health policy and system issues. KTE initiatives and incorporating HPSR with policy are more likely to involve those who provide support to high level policy-makers involved in the policy development process, as they remain in their post for longer periods of time, have the organisational memory and are typically more experienced with HPSR. This means that any processes put into place should take into account policy-makers, those that support policy-makers, and senior executives involved in the policy development process in Israel. More infrastructures and processes to support KTE in Israel need to be put into place, taking into consideration the viewpoints of the stakeholders involved [4, 5].

In two separate studies, Ellen et al. [4, 5] explored the views of both health systems and policy researchers and health policy-makers in Israel regarding the role of HPSR in health policy-making. Researchers conducting HPSR in Israel may be based in research institutes, academic institutions, government agencies, the four health insurance funds, hospital settings, or have appointments at a combination of these. The National Institute for Health Policy Research is an independent association charged by the Israeli Health Council, a body appointed by law to advise and assist the MOH in certain matters. It is the primary organisation involved in directing and funding HPSR at the national level. The money for the grants it provides comes from a budget set aside for such research by the MOH. The Myers-JDC-Brookdale Institute is a partnership between the Israeli government, the American Jewish Joint Distribution Committee and the Myers Foundation, and is also a major player, conducting studies especially related to societal issues. Some of the health maintenance organizations also have research institutes which conduct HPSR. Many researchers that have appointments within these institutes also have academic appointments as well.

Ellen et al.’s [4, 42] work revealed that fewer than half of these researchers were involved in KTE activities, which includes interacting with policy-makers, and fewer than half were engaging in bridging activities to facilitate the use of their research by their target audience. Policy-makers are the other side of the equation as knowledge users. Previous research in this area demonstrated that data usage increased over time, there were instances where key data was missing and, moreover, policy-makers rarely explored how to use data to contribute to their decision-making in a systematic way. Political motives have also been implicated as a strong driver that informs policy-making.

To our knowledge, our work was the first in Israel to examine the views on the use of HPSR by health system policy-makers and researchers in a quantitative manner. The purpose of this study was to compare the perceptions of Israeli health systems and policy researchers and health services policy-makers regarding the role of HPSR, factors influencing its uses and potential facilitators and barriers to HPSR, and implementation of KTE activities.

## Methods

This study builds on previous work examining the perceptions of health systems and policy researchers and of health policy-makers, and a full description of the methods appear elsewhere [4, 5].

### Developing the survey

The survey used was based on a pre-existing, tested survey showing high internal consistency and good face and content validity [43] based on earlier surveys that were conducted in this field [13, 44]. The original lengthy version was modified and adapted to the Israeli setting. The modifications to the survey were performed in conjunction with and approved by one of the co-authors of the original survey. Although some questions were modified to fit the Israeli context, or removed due to the length of the survey, those modifications were made by experts either in KTE or Israeli health policy. The survey consisted of demographics and quantitative sections. The demographics section consisted of questions related to sex, age, degrees completed, primary affiliation and main research or policy domain (depending on the group). The quantitative sections consisted of seven main sections, all answered on a 5-point scale ranging from 1 (strongly disagree) to 5 (strongly agree), which are detailed in Box 1. No identifiers were linked with the data.

### Selecting and recruiting the participants

Due to the size of the country and the size of this field of research and policy in Israel, and after consulting with senior officials in both the research and policy setting, it was estimated that there were approximately 60–100 health systems and policy researchers and 60 health policy-makers in Israel. Therefore, due to the manageable size of potential respondents, it was decided that all of them would be invited to participate in the survey.

Health systems and policy researchers from academic institutions, hospital settings, government agencies, the four health insurance funds, and research institutes were invited to participate in the survey. These researchers were located using publicly available websites related to the institutions mentioned above as well as the list of research projects that were funded by the National Institute for Health Policy Research in Israel. For the purpose of identifying survey participants, a broad definition of health systems and policy research was applied, i.e. research related to ‘governance, financial and delivery arrangements for health care and population health services. Respondents were asked to reply to survey questions with this definition in mind. In recruiting the researchers, a preliminary letter describing the purpose of the study was e-mailed to all potential respondents in both English and Hebrew, containing a link to the survey. Non-respondents were sent reminders 2 and 6 weeks after the first reminder. Each potential participant received a phone call informing them of the survey and reminding them to complete the online portion. Other means of increasing the response rate identified in a systematic review were used as well [45] and are further detailed in Ellen et al. [5].

Health policy-makers that are directly involved in health policy-making as well as those who support it were invited to participate in this research. Our definition of policy-maker included those who are involved directly in health policy-making as well as those who support these processes (i.e. those who consult with policy-makers or support them with relevant information, such as CEOs, managers or heads of different governmental departments and councils). The list of potential participants included officials from the Knesset, Israel’s MOH, Ministry of Finance, health services organisations and other organisations (Hospital CEOs, CEO of the National Insurance Institute of Israel, Israel Medical Association; members of the Knesset). Their areas of HPSR could encompass any field of health policy research, including public health, funding, administration and governance, etc. Eligible participants were those who had been involved in at least one health policy-making process in the Israeli health system in the last 5 years. They were identified using publicly available websites (e.g. Israel’s MOH), consultations with leaders in the health policy field familiar with health policy-making in Israel, the National Institute for Health Policy, and a respondent-driven sampling technique. Policy-makers were invited to participate via e-mail using a letter describing the purpose of the study and inviting recipients to participate. A reminder was sent out 2 weeks subsequent to the initial request followed by another reminder sent a month later. Potential participants who did not reply to the third reminder were telephoned. The researchers completed the survey using a web-based tool, while the policy-makers completed the survey during a face-to-face meeting.

### Data analysis

Quantitative responses for both groups were exported to the Statistical Package for Social Sciences (SPSS) and they were analysed using descriptive statistics. Descriptive analyses were carried out for closed-ended questions in both groups. We combined the two highest response options (strongly agree and agree) and the two lowest response options (strongly disagree and disagree) for close-ended questions; the percent of respondents who gave the two highest response options are presented in the tables. A more complete presentation of the responses is available in Additional file 1. We also compared the survey responses from each group to each other for the responses on sections 1 through 6 using the χ^2^ test for differences between researchers and policy-makers. No formal hypotheses were made and the statistics were mainly used to highlight possible differences not plainly visible. The χ^2^ values presented should be considered descriptive in nature, and as indication of possible areas for future in depth scrutiny.

### Ethics

Ethics approval was received from the Jerusalem College of Technology’s (the first author’s primary affiliation) sub-committee on ethics.

## Results

A total of 107 health policy and systems researchers were invited to participate in the survey, of whom 37 responded (response rate of 35%). Among the 37 respondents, 16 were males and 17 females. The average age of the respondents was 51.9 (SD 10.9) years with a range of 30 to 68 years; 16 respondents (44.4%) were affiliated with an academic university, 12 (33.3%) with a research institute not within a university, 7 (19.4%) work in a teaching hospital setting, and one was affiliated with a government department or agency. Overall, 73 potential policy-maker respondents were contacted; three of them did not meet the criteria for participation in the study (e.g. career change or current area of work does not focus on health systems and policy).

A total of 32 policy-makers were interviewed for a response rate of 46%. Among the 32 respondents, 23 were males and 9 females. The average age of the respondents was 54.7 (SD 11.3) years with a range of 34 to 83 years; 18 (56%) participants were from the Ministry of Health, 4 (12.5%) each from health service organisations and national councils, and the remainder from other organisations such as the national insurance institute, the Ministry of Finance and members of the Knesset; 5 (16%) of the respondents were either Ministers, current or past director generals of the MOH or CEOs, 9 (28%) were deputy director-generals or vice-presidents, and 18 (56%) were in other positions, i.e. department heads or chairpersons of national committees.

There were a few differences in the perceptions of the groups regarding the role of HPSR and the factors that influence its use (Table 1). While only a little more than two-thirds of the researchers felt that use of evidence from HPSR in policy was hindered by practical constraints to implementation (such as financial implications), 91% of the policy-makers perceived that those constraints hindered the use of HPSR evidence in policy. Further, 59% of the researchers felt that the use of evidence from HPSR in policy-making was hindered by a lack of coordination between knowledge producers and knowledge users, while less than one-third of the policy group (32%) felt this to be true.

**Table 1:** The role of health policy and systems research (HPSR) and the factors that influence its use by health policy-makers and stakeholders in Israel

	Researchers	Policy-makers	χ^2^ test for independence
	Percentage agree or strongly agree		χ^2^(1)
Use of evidence from HPSR in policy was hindered by practical constraints to implementation such as financial implications	68	91	5.208^*^
Evidence from HPSR does help raise health policy-makers and stakeholders’ awareness on policy issues	65	49	0.199
Lack of coordination between policy-makers and researchers hindered the use of evidence from HPSR in the health policy-making process	59	32	4.605^*^
Evidence from HPSR does help health policy-makers and stakeholders to identify and/or choose policy alternatives	54	63	0.464
Use of evidence from HPSR in policy was hindered by a non-receptive policy environment	47	34	1.097
Use of evidence from HPSR in policy was hindered by findings that were politically sensitive or were inconsistent with a policy direction	47	52	0.135
Evidence from HPSR was presented to policy-makers and stakeholders in a timely manner and in a format that they can understand	34	25	0.560

^*^p < 0.05

A higher percentage of policy-makers, as compared to researchers, reported that facilitators in the KTE process are in place (Table 2). Specifically, 68% of the policy-makers felt they have access to technical support for acquiring, assessing and applying HPSR research while only 42% of the research group felt that the policy-makers had such access. Additionally, 68% indicated that structures and processes exist to link them with researchers to facilitate the use of HPSR in policy while only 38% of researchers agreed. Policy-makers were more convinced that national funding sources encourage KTE activities, as 70% of them reported this as a facilitator while only 38% of the researchers were in agreement. Similarly, 45% of the policy-makers saw themselves as creating opportunities to develop joint HPSR research initiatives, while only 22% of researchers viewed this to be true.

**Table 2:** Potential facilitators and barriers to the use and implementation of knowledge transfer and exchange (KTE) activities

Factors	Researchers	Policy-makers	χ^2^ test for independence
	Percentage agree or strongly agree		χ^2^(1)
Facilitators			
National funders formulate their priorities and calls for proposals in response to national and regional needs	59	78	2.376
Personal and organisational contacts among policy-makers were quite stable over time	43	61	1.946
Funding sources (e.g. granting agencies) consider KTE activities an allowable expense	43	65	3.001
Policy-makers have access to technical support for acquiring, assessing and applying health policy and systems research (HPSR)	42	68	4.555^*^
Structures and processes exist to link you with policy-makers	38	68	6.039^*^
National funding sources encourage KTE activities	38	70	6.869^**^
Policy-makers invest financial and/or human resources in KTE activities	22	42	3.261
Policy-makers create opportunities to develop joint HPSR research initiatives with them	22	45	4.271^*^
Barriers			
Policy-makers lack the expertise for acquiring, assessing and applying HPSR research	59	31	5.274^*^
Priorities in the health system draw attention away from HPSR research	59	43	1.727
Policy-makers do not make decisions on the basis of HPSR research	51	24	5.043^*^
Policy-makers do not have technical access (i.e. journal subscriptions, links to research) to the appropriate databases to search for HPSR research	32	10	4.798^*^

^*^p < 0.05; ^**^p < 0.01

A higher percentage of the researchers perceived barriers within the KTE environment as compared to the policy group (Table 2), wherein 59% viewed policy-makers’ lack of expertise for acquiring, assessing and applying HPSR research as a barrier to the use and implementation of KTE while only 31% of policy-makers saw this as a barrier. Further, 51% of the research group felt that policy-makers do not make decisions based on HPSR as compared to 24% of policy-makers who shared this view. Close to one-third of the researchers identified policy-makers’ lack of access to appropriate databases to search for HPSR as a barrier while only 10% of the policy-makers agreed.

Although there were differences in the group responses regarding additional facilitators and barriers at the level of organisational support for KTE activities, these differences were found to be less pronounced (Table 3).

**Table 3:** Additional facilitators and barriers at the level of organisational support for knowledge transfer and exchange (KTE) activities

	Researchers	Policy-makers	χ^2^ test for independence
	Percentage agree or strongly agree		χ^2^(1)
KTE was hampered by a lack of incentives for KTE activities within organisations that conduct health policy and systems research (HPSR)	38	15	3.772
Organisations that conduct HPSR made financial and human resources available to assist with KTE activities	24	46	3.056
Organisations that conduct HPSR were not seen as a credible source of research	14	7	0.673

Results of the surveys indicated that researchers and policy-makers mostly shared similar views with regards to the research available to knowledge users (Table 4). One obvious difference was with respect to 14% of researchers reporting that available research lacked credibility among target audiences, while none of the policy-makers agreed.

**Table 4:** Alignment of available research to needs of knowledge users

	Researchers	Policy-makers	χ^2^ test for independence
	Percentage agree or strongly agree		χ^2^(1)
Available research coincided with the needs and expectations of target audiences	51	37	1.291
Available research coincided with my country’s priorities (e.g. with a National Research Agenda)	43	48	0.152
Available research was not considered relevant by policy-makers	28	11	2.617
Available research lacked credibility among target audiences	14	0	4.099^*^
No research was ready for use	5	4	0.064

^*^p < 0.05

Survey results showed large differences in the perceptions of the two groups regarding one aspect of policy formulation, wherein 62% of the researchers felt that policy formulation is usually based on internal MOH discussions and ad hoc processes while only 34% of the policy-makers agreed (Table 5).

**Table 5:** Factors that influence health policy-making in Israel

	Researchers	Policy-makers	χ^2^ test for independence
	Percentage agree or strongly agree		χ^2^(1)
Broad challenges in intergovernmental (i.e. Ministry of Health, Ministry of Finance) relations hindered the health policy-making process	76	91	2.669
Broad challenges in government/provider relations hindered the health policy-making process	69	59	0.752
Policy formulation is usually based on internal Ministry of Health discussions and ad hoc process rather than evidence-based processes	62	34	5.301^*^

^*^p < 0.05

Survey results indicated differing views regarding the organisations they perceived as exerting a strong influence on health policy-making, wherein 89% of the researchers viewed physician associations as exerting a strong influence, as compared to 59% of the policy-makers who agreed with this view. While 88% of the researchers viewed limited health funding as a strong influence on the health policy-making process, all (100%) of the policy-makers viewed it as such. Further, 22% of researchers reported that donor organisations exert a strong influence on the policy-making process as compared to only 3% of policy-makers who agreed (Table 6).

**Table 6:** Groups or factors that exert a strong influence on the health policy-making process

	Researchers	Policy-makers	χ^2^ test for independence
	Percentage agree or strongly agree		χ^2^(1)
Health insurance funds	92	77	2.663
Physician associations	89	59	7.870^**^
Limited health funding (the economy)	88	100	4.008^*^
Media	69	71	0.018
Values of governing parties	61	41	2.846
Public opinion	53	38	1.594
Nursing associations	46	31	1.473
Research about problems related to healthcare or health systems	39	19	3.035
Other countries’ health policies	31	35	0.183
Donor organisations	22	3	5.380^*^
Other types of health professional associations	22	20	0.048

^*^p < 0.05, ^**^p < 0.01

Similar to the χ^2^ results, a larger percentage of respondents from the policy group perceived KTE activities were in place as compared to the researcher group (Table 7); 65% of the policy-makers reported receiving copies of articles and/or systematic reviews about HPSR and 55% reported receiving mailings or e-mails tailored to their needs from researchers, while only a quarter of the researchers reported providing these items. While over three-quarters of the policy-makers indicated receipt of or access to a searchable database of articles/systematic HPSR reviews, only 9% of the researchers indicated providing access to such information. A higher percentage of policy-makers reported receiving training from researchers on developing their capacity to use HPSR than researchers reported providing. A higher proportion of policy-makers also indicated maintaining long-term contact with researchers as compared with the researchers’ perception regarding maintaining contact with policy-makers.

**Table 7:** Engagement with knowledge transfer and exchange (KTE) activities

Researchers	Percentage frequently or always		Policy-makers
Provided copies of articles and/or systematic reviews about health policy and systems research (HPSR) to policy- and/or decision-makers	26	65	Received copies of articles and/or systematic reviews about HPSR from policy- and/or decision-makers
Provided mailings or e-mails with content tailored to specific policy- and/or decision-makers	26	55	Received mailings or e-mails with content tailored to specific policy- and/or decision-makers
Interacted with credible messengers/sources outside your organisation to promote HPSR	25	68	Interacted with credible messengers/sources outside your organisation to obtain HPSR
Provided articles, reports, syntheses, formal systematic reviews and/or messages to policy- and/or decision-makers without an explicit request	21	42	Received articles, reports, syntheses, formal systematic reviews and/or messages from policy- and/or decision-makers without an explicit request
Interacted with policy- and/or decision-makers when developing a specific research question, objectives or hypothesis	35	52	Interacted with researchers when developing a specific research question, objectives or hypothesis
Interacted with policy- and/or decision-makers through events organised by them or their organisation or through informal conversations	38	71	Interacted with researchers through events organised by them or their organisation or through informal conversations
Assessed or participated in assessments of the usefulness and impact of your KTE activities	12	47	Assessed or participated in assessments of the usefulness and impact of your KTE activities
Provided access to a searchable database of articles, reports, syntheses and/or formal systematic reviews on HPSR	9	77	Received/had access to a searchable database of articles, reports, syntheses and/or formal systematic reviews on HPSR
Provided training to policy- and/or decision-makers to develop their capacity to acquire, assess, adapt and apply HPSR	12	27	Received training from policy- and/or decision-makers to develop capacity to acquire, assess, adapt and apply HPSR
Established and/or maintained long-term partnerships with HPSR policy- and/or decision-makers (e.g. through an advisory board)	29	67	Established and/or maintained long-term partnerships with HPSR researchers (e.g. through an advisory board)

## Discussion

### Summary of study findings

This study examined differences in the perception of the KTE process between knowledge producers (researchers) and knowledge users (policy-makers). While some perceptions were aligned, there were differences, e.g. a higher percentage of researchers perceived that policy-makers do not make decisions based on HPSR. Although policy-makers may be open to using evidence to influence policy, it may be challenging to implement their plans in a real world setting as is evidenced by their indication that practical constraints hinder the incorporation into policy-making. Fewer researchers agreed that practical constraints pose a hindrance, which may be because they are not usually involved with implementation or they do not frequently interface with political and financial bodies and may therefore be less aware of practical considerations. Policy-makers may view research evidence as just a fraction of all that contributes to policy-making and that the proportion of their efforts to incorporate research relative to other tasks is appropriate.

A higher percentage of researchers reported the lack of coordination between knowledge producers and users as an impediment to the use of research in policy-making. When there is a lack of coordination it is more likely to be noted by the researchers. HPSR can contribute to one or more of the areas of activity in policy-making, including policy agenda setting, formulation and implementation [46]. Policy-makers plan policies regardless of collaboration with researchers and it is possible that they use research evidence in one of these areas but not in policy implementation. As the research-based underpinnings of policy may not be apparent when a policy is implemented, the perception of the researcher is that it is not being used. In studies attempting to reveal the level of research utilisation in policy, “*examples suggest there is a greater level of utilisation and final outcomes in terms of health, health equity, and social and economic gain than is often assumed, whilst still showing much underutilisation*” [46]. This explains that there is variation in the degree of utilisation, within and between studies. As the use of their research might not be apparent to the researchers, they could be underreporting the amount of KTE efforts. A greater feeling of involvement and sense of mutuality on the policy-makers’ side can also be noted in the responses presented in Table 7. Alternatively, it is possible that the interaction between policy-makers and researchers is confined to a relatively small group within the researchers’ community, leaving the rest unaware of this joint work.

Policy-makers tended to take a more positive view on their access to technical support for acquiring, assessing and applying research, e.g. 77% of policy-makers claimed that they received or had access to a searchable database on HPSR, while only 9% of researchers claimed they provided such access. While this may be explained by policy-makers gaining access through sources other than researchers, it can also indicate that either researchers are not sufficiently aware of the resources available, or that policy-makers overestimate those resources, either in quantity or in quality. There are a number of databases, of varying scope and degree of inclusiveness, for research supporting health policy-making. While there is considerable discussion in the literature on the type of study (i.e. qualitative vs. quantitative, use of systematic review, etc.) the issue of where to initially search for evidence is less addressed. In a study of Australian drug policy-makers [47], Ritter states that the internet, including specialist websites and generic search engines such as Google, was the third most common source reported by respondents. Mays et al. [48] also noted the use of databases (without delineation) and the internet as sources and expanded on the difficulties of performing electronic searches for the types of studies that constitute HPSR, particularly qualitative studies, as well as attempts to improve those searches.

Even when policy-makers can access information, they might face the challenge of understanding and applying HPSR. Other research has noted challenges to policy-makers in using HPSR and the need for HPSR that is more useful to policy-makers [37]. Here, also, more researchers than policy-makers felt that policy-makers lack the expertise for acquiring, assessing and applying HPSR. With research being written by researchers, often for researchers, using scientific concepts and jargon, it may be less suitable to the policy-makers’ understanding. Improving the way research is communicated is a two-way street, wherein policy-makers should improve their expertise in understanding HPSR and researchers should package their research in a more usable way [49]. Policy-makers need to examine numerous aspects in their decision-making process and amalgamate large amounts of information; therefore, methods such as rapid reviews and policy briefs, highlighting relevant conclusions and outcomes, can make using research much easier [50]. Researchers need to package HPSR in ways that are easily understood by policy-makers and that is presented interestingly and convincingly [33, 35, 51, 52].

There are cases where the lack of research uptake has little to do with research quality, skill sets or interactions but rather with strong interests. At times, the political climate and values of the governing parties exert a strong influence as well [35, 38, 51]. In one study, researchers indicated that use of evidence in policy-making was hindered by (among other issues) politically sensitive findings [44] and another study noted that the “*lack of willingness of some policy makers to use research*” was “*greatly influenced by the political context within the country*” [35]. Further, policy-makers have been known to adopt evidence when it supports what they have already decided and will not consider contradicting evidence [36]. Almost half of the researchers in this study had similar sentiments regarding the decision-making process, but, perhaps more intriguingly, so did half of the policy-makers. The differences in perceptions uncovered in our study reveal that communication between the groups is lacking and increased collaboration could be of benefit.

### Strengths/limitations

The biggest strength of this study is that, to our knowledge, it is the first to compare the views of researchers and policy-makers on the use of HPSR in the KTE process in Israel. Furthermore, there are few studies comparing the perceptions of researchers and policy-makers on KTE activity, and ours appears to be the first to do so quantitatively, making it possible to highlight specific aspects where differences may lie. Additional strengths are that close-ended questions were developed based upon a pre-existing and validated instrument [13, 43, 44]. However, no open-ended questions were included, which can be seen as a limitation. The main limitation, however, was that, despite the efforts to recruit participants, the response rate was lower than hoped for, with a risk of self-selection bias. Our small sample size may have had an effect on the statistical significance and interpretation of our results. In addition, the surveys are based on self-reports, thus one cannot exclude social desirability bias. These limitations may affect both internal validity and the application of the findings to the broader population. This study was meant to look for differences between the two groups (researchers and policy-makers). However, there was significant diversity within each of the groups with regards to background, policy area, type of research, etc., all of which can affect their perceptions on the decision-making process and the role of HPSR. While this can give us a wider point of view, it can also result in greater within-group variance that may obscure possible between-group differences. This is both a strength of this study and a limitation. Future research can implement a mixed-methods design to provide a fuller understanding of the factors underpinning the issue.

### Implications and future work

This study provides insight to the differences in the perceptions of researchers and policy-makers on the policy-making process and the different factors that influence it. Understanding the perceptions of both parties within the Israeli context is imperative, as they both play important roles in improving decision-making processes and, as a result, the quality of health policy decisions. Future work should focus on minimising the challenges highlighted in the surveys and generating new KTE activities while taking the differences into account. These activities could include making the researchers aware of the most effective manner in which to package their results, which may vary by context and should be supported by research, providing training to policy-makers and assuring that they have technical access to appropriate databases to search for HPSR. Some practical steps can be taken on the end of both researchers and policy-makers. Researchers can focus on packaging information to users in jargon-free language which highlights practical actions [53, 54] and that is adapted to specific contexts and situations [54, 55]. Actionable messages arising from research must be identified, reworked for different user groups and disseminated to each group in a way that encourages uptake [25]. On the knowledge-user end, the development of tools to aid in finding relevant, useful research can be helpful to the successful translation of research into practice [52, 53, 56].

Perhaps the most effective KTE activity would be to encourage the groups to work together. Collaboration between knowledge producers and knowledge users may result in a clarification of the issues noted, thereby reducing the extent of the gap between the two crucial activities in the evidence to policy process. Relationships between the policy-makers and researchers could result in increasing the use of HPSR in policy [3, 57]. There is much discussion on the benefits of knowledge producers and knowledge users working together [34–37]. Translating research into practice has been found to be influenced by the “*relationship and trust between the researchers and policy makers*” [35]. Uptake into policy has been found to depend on human relationships even over the objectivity of research [54]. Improvement in communication between those conducting research and policy-makers affects the use of evidence in policy-making [6, 37], and therefore programmes encouraging collaboration between researchers and policy-makers could increase research usage in policy formation [36]. In Oliver et al.’s [6] systematic review, collaboration between researchers and policy-makers was one of the two main facilitators of the use of evidence by policy-makers. Researchers and policy-makers may want to create knowledge translation platforms together to encourage dialogue and participation from both sides.

Different collaboration initiatives exist, for example, Langlois et al. [58] showed that evidence was used in policy-making when researcher/policy-maker groups (Policy Buddies) summarised, presented and discussed findings from systematic reviews. In addition, policy-makers reported that Policy Buddies helped them “*recognise the value of research evidence to their daily work*” [58]. Other examples include encouraging knowledge users to be more involved in study design and early stages of research, thus ensuring that the work can answer the needs of the knowledge user [4]. Additionally, structured opportunities to enhance collaboration and build networks should be provided and prioritised by health systems. These might include smaller, periodic meetings, larger conferences, research or journal clubs, and collaborating on committees regarding specific issues. Knowledge translation networks, such as EVIPNet, for example, facilitate evidence-informed policy through networking. Networking enhances mutual learning, increases and diversifies expertise, and promotes communication and problem solving between groups [54].

Although the issues covered here are not new to KTE as a whole, they are new in the Israeli context, as KTE is a relatively new field in this country. KTE activities are not yet entrenched in the culture, and both researchers and policy-makers need to understand the advantages of organised KTE in order to fully benefit from its implementation in the Israeli context. This study’s findings have the potential to provide Israeli policy-makers and researchers the insight they need to work together and to build interventions to support HPSR. Observations from both parties prove that there is strong perceived linkage between policy-makers, researchers and other stakeholders that can aid in transferring research into practice. Linkage and exchange efforts have been proven to occur in environments where there are positive relationships between researchers and policy-makers, as is the case in Israel. Additionally, future KTE initiatives should focus on establishing regular priority-setting processes, funding new research in the form of partnerships between researchers and knowledge users or health services agencies, and ensuring the overall capacity to conduct or commission research.

## Conclusion

To our knowledge, this study is the first to compare, in a quantitative manner, the views of health system policy-makers and researchers on the use of HPSR in Israel. This research demonstrated that there are differences in the perceptions of knowledge producers and knowledge users on factors hindering the implementation of HPSR, the accessibility of evidence to policy-makers, evidence credibility, and groups/factors influencing health policy-making in Israel. The policy-making group responses indicate that they perceive the challenges to be less severe than as perceived by the researchers. Differences in perceptions exist among the many players in translating research into policy. While each country and healthcare system has its unique attributes, some of the points captured here may well be applicable to people involved in KTE in other countries. Campbell et al. [3] noted that different stakeholders “*adopt diverse and often conflicting views*” of evidence because their understanding of the concept seemed to reflect their personal experiences. Further work is needed on creating a shared understanding and expose the groups to new KTE activities, where they can share their perceptions and gain an understanding of each other and the values they each possess. Once the differences in their perceptions are understood, it will provide a foundation upon which to draw conclusions regarding how the two groups can better attempt to improve the KTE process.

## Box 1. Quantitative sections

**Table:** 

Section	Description
1	16 items regarding participants’ views on the barriers and facilitators for KTE
2	3 items regarding the support for KTE within participants’ organisation
3	5 items exploring participants’ views about the research being produced and its possible impact on the policy-making process
4	3 items regarding the factors that influence health policy-making in Israel
5	11 items surveying participants’ views on the groups or factors that could have exerted a strong influence on the health policy-making process
6	7 items focusing on the function of HPSR and the influences on the use of HPSR by health policy-makers and stakeholders in Israel
7	KTE activities in place

## Additional file

Additional file 1: **Table S1.** The role of HPSR and the factors that influence the use of HPSR by health policy-makers and stakeholders in Israel. **Table S2.** Potential facilitators and barriers to the use and implementation of KTE activities. **Table S3.** Additional facilitators and barriers at the level of organisational support for KTE activities. **Table S4.** Alignment of available research to the needs of knowledge users. **Table S5** Factors that influence health policy-making in Israel. **Table S6.** Groups or factors that exert a strong influence on the health policy-making process. **Table S7.** Engagement with KTE activities. (DOCX 58 kb)

## Abbreviations

HPSR: health policy and systems research
KTE: knowledge transfer and exchange
MOH: Ministry of Health

## References

[1] Mitton C, Adair CE, McKenzie E, Patten SB, Perry BW (2007). Knowledge transfer and exchange: review and synthesis of the literature. Milbank Q.

[2] Orton L, Lloyd-Williams F, Taylor-Robinson D, O'Flaherty M, Capewell S (2011). The use of research evidence in public health decision making processes: systematic review. PLoS One.

[3] Campbell DM, Redman S, Jorm S, Cooke M, Zwi AB, Rychetnik L (2009). Increasing the use of evidence in health policy: practice and views of policy makers and researchers. Aust New Zealand Health Policy.

[4] Ellen ME, Horowitz E, Vaknin S, Lavis JN (2016). Views of health system policymakers on the role of research in health policymaking in Israel. Israel J Health Policy Res.

[5] Ellen ME, Lavis JN, Shemer J (2016). Examining the use of health systems and policy research in the health policymaking process in Israel: views of researchers. Health Res Policy Syst.

[6] Oliver K, Innvar S, Lorenc T, Woodman J, Thomas J (2014). A systematic review of barriers to and facilitators of the use of evidence by policymakers. BMC Health Serv Res.

[7] Lavis JN, Davies HTO, Oxman AD, Denis J-L, Golden-Biddle K, Ferlie E (2005). Towards systematic reviews that inform health care management and policy-making. J Health Serv Res Policy.

[8] Sauerborn R, Nitayarumphong S, Gerhardus A (1999). Strategies to enhance the use of health systems research for health sector reform. Tropical Med Int Health.

[9] Grimshaw JM, Eccles MP, Lavis JN, Hill SJ, Squires JE (2012). Knowledge translation of research findings. Implement Sci.

[10] Innvaer S, Vist G, Trommald M, Oxman AD (2002). Health policy-makers’ perceptions of their use of evidence: a systematic review. J Health Serv Res Policy.

[11] First Global Symposium on Health Systems Research. Montreux Statement from the Steering Committee of the First Global Symposium on Health Systems Research. 2011. http://www.who.int/mediacentre/events/meetings/2010/hsr/en/. Accessed 10 July 2018.

[12] El-Jardali F, Ataya N, Jamal D, Jaafar M (2012). A multi-faceted approach to promote knowledge translation platforms in eastern Mediterranean countries: climate for evidence-informed policy. Health Res Policy Syst.

[13] Lavis JN, Guindon GE, Cameron D, Boupha B, Dejman M, Osei EJ (2010). Bridging the gaps between research, policy and practice in low- and middle-income countries: a survey of researchers. CMAJ.

[14] Ellen ME. Knowledge Translation on Ageing and Health: A Framework for Policy Development. World Health Organization. 2012. http://www.who.int/ageing/publications/knowledge_translation_en.pdf. Accessed 10 Oct 2016.

[15] World Health Assembly. Resolution on Health Research. 2011. http://www.who.int/rpc/meetings/58th_WHA_resolution.pdf. Accessed 4 Apr 2013.

[16] World Health Organization (2004). The Mexico Statement on Health Research: Knowledge for Better Health: Strengthening Health Systems.

[17] World Health Organization (2006). Bridging the “Know-Do” Gap.

[18] Canadian Institute for Health Information. More About Knowledge Translation at CIHR. 2012. http://www.cihr-irsc.gc.ca/e/29418.html. Accessed 10 July 2018.

[19] Straus SE, Tetroe JM, Graham I (2011). Knowledge translation is the use of knowledge in health care decision making. J Clin Epidemiol.

[20] Tabak RG, Khoong EC, Chambers DA, Brownson (2012). Bridging research and practice: models for dissmemination and implementation research. Am J Prev Med.

[21] Nicolini D, Powell J, Conville P, Martinez-Solano L (2008). Managing knowledge in the healthcare sector: a review. Int J Manag Rev.

[22] Graham I, Tetroe J (2007). Some theoretical underpinnings of knowledge translation. Acad Emerg Med.

[23] Kitson A, Harvey G, McCormack B (1998). Enabling the implementation of evidence based practice: a conceptual framework. Qual Health Care.

[24] Dobbins M, Ciliska D, Cockerill R, Barnsley J, DiCenso A (2002). A framework for the dissemination and utilization of research for health-care policy and practice. Online J Knowl Synth Nurs.

[25] Lavis JN, Lomas J, Hamid M, Sewankambo NK (2006). Assessing country-level efforts to link research to action. Bull World Health Organ.

[26] Graham ID, Logan J, Harrison MB, Straus SE, Tetroe J, Caswell W (2006). Lost in knowledge translation: time for a map?. J Contin Educ Heal Prof.

[27] Graham ID, Logan J (2004). Innovations in knowledge transfer and continuity of care. Can J Nurs Res.

[28] Jones N, Datta A, Jones H (2009). Knowledge, Policy and Power: Six Dimensions of the Knowledge–Development Policy Interface.

[29] Nash R, Hudson A, Luttrell C (2006). Mapping political context: a toolkit for civil society Organisations.

[30] Rycroft-Malone J, Seers K, Titchen A, Harvey G, Kitson A, McCormack B (2004). What counts as evidence in evidence-based practice?. J Adv Nurs.

[31] Ellen ME, Lavis JN, Ouimet M, Grimshaw J, Bedard PO (2011). Determining research knowledge infrastructure for healthcare systems: a qualitative study. Implement Sci.

[32] Ellen ME, Panisset U, de Carvalho IA, Goodwin J, Beard J (2017). A knowledge translation framework on ageing and health. Health Policy.

[33] Petticrew M, Whitehead M, Macintyre SJ, Graham H, Egan M (2004). Evidence based public health policy and practice: evidence for public health policy on inequalities: 1. The reality according to policymakers. J Epidemiol Community Health.

[34] Whitehead M, Petticrew M, Graham H, Macintyre SJ, Bambra C, Egan M (2004). Evidence for public health policy on inequalities: 2: assembling the evidence jigsaw. J Epidemiol Community Health.

[35] Uzochukwu B, Onwujekwe O, Mbachu C, Okwuosa C, Etiaba E, Nystrom ME (2016). The challenge of bridging the gap between researchers and policy makers: experiences of a health policy research group in engaging policy makers to support evidence informed policy making in Nigeria. Glob Health.

[36] Choi BCK, Pang T, Lin V, Puska P, Sherman G, Goddard M (2005). Can scientists and policy makers work together?. J Epidemiol Community Health.

[37] Shroff Z, Aulakh B, Gilson L, Agyepong IA, El-Jardali F, Ghaffar A (2015). Incorporating research evidence into decision-making processes: researcher and decision-maker perceptions from five low-and middle-income countries. Health Res Policy Syst.

[38] El-Jardali F, Lavis JN, Ataya N, Jamal D, Ammar W, Raouf S (2012). Use of health systems evidence by policymakers in eastern Mediterranean countries: views, practices, and contextual influences. BMC Health Serv Res.

[39] Jonsson K, Tomson G, Jonsson C, Kounnavong S, Wahlstrom R (2007). Health systems research in Lao PDR: capacity development for getting research into policy and practice. Health Res Policy Syst.

[40] Tomson G, Paphassarang C, Jonsson K, Houamboun K, Akkhavong K, Wahlstrom R (2005). Decision-makers and the usefulness of research evidence in policy implementation: a case study from Lao PDR. Soc Sci Med.

[41] Rosen B. The Israeli Health Care System (chapter in 2015 International Profiles of Health Care Systems). Edited by Mossialos E, Wenzl M, Osborn R, Sarnak D. 2016. The Commonwealth Fund. http://www.commonwealthfund.org/~/media/files/publications/fund-report/2016/jan/1857_mossialos_intl_profiles_2015_v7.pdf. Accesed 4 Apr 2016.

[42] Ellen ME, Lavis JN, Sharon A, Shemer J (2014). Health systems and policy research evidence in health policy making in Israel: what are researchers’ practices in transferring knowledge to policy makers?. Health Res Policy Syst.

[43] Cameron D, Lavis JN, Guindon GE, Akhtar T, Becerra-Posada F, Ndossi GD (2010). Bridging the gaps among research, policy and practice in ten low- and middle-income countries: development and testing of a questionnaire for researchers. Health Res Policy Syst.

[44] El-Jardali F, Lavis J, Ataya N, Jamal D (2012). Use of health systems and policy research evidence in the health policymaking in eastern Mediterranean countries: views and practices of researchers. Implement Sci.

[45] Edwards PJ, Roberts I, Clarke MJ, DiGuiseppi C, Wentz R, Kwan I, et al. Methods to increase response to postal and electronic questionnaires. Cochrane Database Syst Rev. 2009;(3):MR000008. 10.1002/14651858.MR000008.pub4. 10.1002/14651858.MR000008.pub4 PMC8941848 19588449

[46] Hanney SR, Gonzalez Block MA, Buxton MJ, Kogan M (2003). The utilization of health research in policy making: concepts, examples and methods of assessment. Health Res Policy Syst.

[47] Ritter A (2009). How do drug policy makers access research evidence?. Int J Drug Policy.

[48] Mays N, Pope C, Popay J (2005). Systematically reviewing qualitative and quantitative evidence to inform management and policy-making in the health field. J Health Serv Res Policy.

[49] Brownson RC, Royer C, Ewing R, McBride TD (2006). Researchers and policymakers: travelers in parallel universes. Am J Prev Med.

[50] Chambers D, Wilson PM, Thompson CA, Hanbury A, Farley K, Light K (2011). Maximizing the impact of systematic reviews in health care decision making: a systematic scoping review of knowledge-translation resources. Milbank Q.

[51] Nabyonga Orem J, Mafigiri DK, Marchal B, Ssengooba F, Macq J, Criel B (2012). Research, evidence and policymaking: the perspectives of policy actors on improving uptake of evidence in health policy development and implementation in Uganda. BMC Public Health.

[52] Ellen ME, Lavis JN, Wilson MG, Grimshaw J, Haynes RB, Ouimet M (2014). Health system decision makers’ feedback on summaries and tools supporting the use of systematic reviews: a qualitative study. Evid Policy.

[53] Lavis JN, Catallo C, Permanand G, Zierler A, BRIDGE Study Team (2011). BRIDGE Summary 1 – Communicating Clearly: Enhancing Information-Packaging Mechanisms to Support Knowledge Brokering in European Health Systems.

[54] EVIPNet Europe (2017). Introduction to EVIPNet Europe: Conceptual Background and Case Studies.

[55] Nutley S, Walter I, Davies HTO (2003). From knowing to doing: a framework for understanding the evidence-into-practice agenda. Evaluation.

[56] Gold M, Fries Taylor E (2007). Moving research into practice: lessons from the US Agency for Healthcare Research and Quality's IDSRN program. Implement Sci.

[57] Ellen ME, Brown AD (2016). Transferring research from researchers to knowledge users: the importance of relationships and getting them right. J Health Serv Res Policy.

[58] Langlois EV, Montekio VB, Young T, Song K, Alcalde-Rabanal J, Tran N (2016). Enhancing evidence informed policymaking in complex health systems: lessons from multi-site collaborative approaches. Health Res Policy Syst.

