# Supplementary material for: How is the use of research evidence in health policy perceived? A comparison between the reporting of researchers and policy-makers
Source: Health Res Policy Syst. 2018 Jul 20;16:64. doi: 10.1186/s12961-018-0345-6 (PMC6053732; doi:10.1186/s12961-018-0345-6)
Supplement: Supplementary file 1 — Table S1. The role of HPSR and the factors that influence the use of HPSR by health policy-makers and stakeholders in Israel. Table S2. Potential facilitators and barriers to the use and implementation of KTE activities. Table S3. Additional facilitators and barriers at the level of organisational support for KTE activities. Table S4. Alignment of available research to the needs of knowledge users. Table S5 Factors that influence health policy-making in Israel. Table S6. Groups or factors that exert a strong influence on the health policy-making process. Table S7. Engagement with KTE activities. (DOCX 58 kb) [file 12961_2018_345_MOESM1_ESM.docx]

**Additional File 1**

**Table S1: The role of HSPR and the factors that influence the use of HSPR by health policymakers and stakeholders Israel**

|  | | **Researchers** | | **Policy Makers** | |
| --- | --- | --- | --- | --- | --- |
|  |  | **N** | **%** | **N** | **%** |
| Use of evidence from HSPR in policy was hindered by practical constraints to implementation such as financial implications | Strongly Disagree | 0 | 0.0% | 0 | 0.0% |
|  | Disagree | 1 | 2.9% | 0 | 0.0% |
|  | Neither agree nor disagree | 10 | 29.4% | 3 | 9.4% |
|  | Agree | 20 | 58.8% | 18 | 56.2% |
|  | Strongly Agree | 3 | 8.8% | 11 | 34.4% |
|  | *Total* | *34* | *100.0%* | *32* | *100.0%* |
| Evidence from HSPR does help raise health policy makers and stakeholders' awareness on policy issues | Strongly Disagree | 0 | 0.0% | 0 | 0.0% |
|  | Disagree | 4 | 11.8% | 1 | 3.1% |
|  | Neither agree nor disagree | 8 | 23.5% | 12 | 37.5% |
|  | Agree | 18 | 52.9% | 13 | 40.6% |
|  | Strongly Agree | 4 | 11.8% | 6 | 18.8% |
|  | *Total* | *34* | *100.0%* | *32* | *100.0%* |
| Lack of coordination between policy makers and researchers hindered the use of evidence from HSPR in the health policymaking process | Strongly Disagree | 0 | 0.0% | 0 | 0.0% |
|  | Disagree | 4 | 11.8% | 12 | 38.7% |
|  | Neither agree nor disagree | 10 | 29.4% | 9 | 29.0% |
|  | Agree | 17 | 50.0% | 6 | 19.4% |
|  | Strongly Agree | 3 | 8.8% | 4 | 12.9% |
|  | *Total* | *34* | *100.0%* | *31* | *100.0%* |
| Evidence from HSPR does help health policy makers and stakeholders to identify and/ or choose policy alternatives | Strongly Disagree | 0 | 0.0% | 0 | 0.0% |
|  | Disagree | 4 | 11.4% | 5 | 15.6% |
|  | Neither agree nor disagree | 12 | 34.3% | 7 | 21.9% |
|  | Agree | 16 | 45.7% | 17 | 53.1% |
|  | Strongly Agree | 3 | 8.6% | 3 | 9.4% |
|  | *Total* | *35* | *100.0%* | *32* | *100.0%* |
| Use of evidence from HSPR in policy was hindered by a non- receptive policy environment | Strongly Disagree | 0 | 0.0% | 6 | 18.8% |
|  | Disagree | 2 | 5.9% | 5 | 15.6% |
|  | Neither agree nor disagree | 16 | 47.1% | 10 | 31.3% |
|  | Agree | 15 | 44.1% | 6 | 18.8% |
|  | Strongly Agree | 1 | 2.9% | 5 | 15.6% |
|  | *Total* | *34* | *100.0%* | *32* | *100.0%* |
| Use of evidence from HSPR in policy was hindered by findings that were politically sensitive or were inconsistent with a policy direction | Strongly Disagree | 0 | 0.0% | 2 | 6.5% |
|  | Disagree | 5 | 14.7% | 8 | 25.8% |
|  | Neither agree nor disagree | 13 | 38.2% | 5 | 16.1% |
|  | Agree | 13 | 38.2% | 11 | 35.5% |
|  | Strongly Agree | 3 | 8.8% | 5 | 16.1% |
|  | *Total* | *34* | *100.0%* | *31* | *100.0%* |
| Evidence from HSPR was presented to policy makers and stakeholders in a timely manner and in a format that they can understand | Strongly Disagree | 0 | 0.0% | 2 | 6.5% |
|  | Disagree | 8 | 22.9% | 9 | 29.0% |
|  | Neither agree nor disagree | 15 | 42.9% | 12 | 38.7% |
|  | Agree | 9 | 25.7% | 7 | 22.6% |
|  | Strongly Agree | 3 | 8.6% | 1 | 3.2% |
|  | *Total* | *35* | *100.0%* | *31* | *100.0%* |

**Table S2: Potential facilitators and barriers to the use and implementation of KTE activities**

|  | | **Researchers** | | **Policy Makers** | |
| --- | --- | --- | --- | --- | --- |
|  |  | **N** | **%** | **N** | **%** |
| **Facilitators** | | | | | |
| National funders formulate their priorities and calls for proposals in response to national and regional needs. | Strongly Disagree | 0 | 0.0% | 0 | 0.0% |
|  | Disagree | 5 | 13.5% | 4 | 14.8% |
|  | Neither agree nor disagree | 10 | 27.0% | 2 | 7.4% |
|  | Agree | 18 | 48.6% | 16 | 59.3% |
|  | Strongly Agree | 4 | 10.8% | 5 | 18.5% |
|  | *Total* | *37* | *100.0%* | *27* | *100.0%* |
| Personal and organizational contacts among policymakers were quite stable over time | Strongly Disagree | 2 | 5.4% | 0 | 0.0% |
|  | Disagree | 6 | 16.2% | 2 | 7.1% |
|  | Neither agree nor disagree | 13 | 35.1% | 9 | 32.1% |
|  | Agree | 14 | 37.8% | 13 | 46.4% |
|  | Strongly Agree | 2 | 5.4% | 4 | 14.3% |
|  | *Total* | *37* | *100.0%* | *28* | *100.0%* |
| Funding sources (e.g., granting agencies) consider KTE activities an allowable expense | Strongly Disagree | 1 | 2.7% | 0 | 0.0% |
|  | Disagree | 9 | 24.3% | 3 | 11.5% |
|  | Neither agree nor disagree | 11 | 29.7% | 6 | 23.1% |
|  | Agree | 15 | 40.5% | 10 | 38.5% |
|  | Strongly Agree | 1 | 2.7% | 7 | 26.9% |
|  | *Total* | *37* | *100.0%* | *26* | *100.0%* |
| Policymakers have access to technical support for acquiring, assessing, and applying HSPR research | Strongly Disagree | 1 | 2.8% | 2 | 6.5% |
|  | Disagree | 10 | 27.8% | 2 | 6.5% |
|  | Neither agree nor disagree | 10 | 27.8% | 6 | 19.4% |
|  | Agree | 14 | 38.9% | 15 | 48.4% |
|  | Strongly Agree | 1 | 2.8% | 6 | 19.4% |
|  | *Total* | *36* | *100.0%* | *31* | *100.0%* |
| Structures and processes exist to link you with policymakers | Strongly Disagree | 4 | 10.8% | 2 | 6.5% |
|  | Disagree | 6 | 16.2% | 6 | 19.4% |
|  | Neither agree nor disagree | 13 | 35.1% | 2 | 6.5% |
|  | Agree | 10 | 27.0% | 14 | 45.2% |
|  | Strongly Agree | 4 | 10.8% | 7 | 22.6% |
|  | *Total* | *37* | *100.0%* | *31* | *100.0%* |
| National funding sources encourage KTE activities. | Strongly Disagree | 2 | 5.4% | 0 | 0.0% |
|  | Disagree | 11 | 29.7% | 3 | 10.0% |
|  | Neither agree nor disagree | 10 | 27.0% | 6 | 20.0% |
|  | Agree | 12 | 32.4% | 14 | 46.7% |
|  | Strongly Agree | 2 | 5.4% | 7 | 23.3% |
|  | *Total* | *37* | *100.0%* | *30* | *100.0%* |
| Policymakers invest financial and/or human resources in KTE activities | Strongly Disagree | 5 | 13.5% | 0 | 0.0% |
|  | Disagree | 15 | 40.5% | 6 | 19.4% |
|  | Neither agree nor disagree | 9 | 24.3% | 12 | 38.7% |
|  | Agree | 8 | 21.6% | 12 | 38.7% |
|  | Strongly Agree | 0 | 0.0% | 1 | 3.2% |
|  | Total | 37 | 100.0% | 31 | 100.0% |
| Policymakers create opportunities to develop joint HSPR research initiatives with them | Strongly Disagree | 5 | 13.5% | 0 | 0.0% |
|  | Disagree | 15 | 40.5% | 10 | 32.3% |
|  | Neither agree nor disagree | 9 | 24.3% | 7 | 22.6% |
|  | Agree | 8 | 21.6% | 12 | 38.7% |
|  | Strongly Agree | 0 | 0.0% | 2 | 6.5% |
|  | *Total* | *37* | *100.0%* | *31* | *100.0%* |
| **Barriers** | | | | | |
| Policymakers lack the expertise for acquiring, assessing, and applying HSPR research | Strongly Disagree | 1 | 2.7% | 4 | 13.8% |
|  | Disagree | 7 | 18.9% | 6 | 20.7% |
|  | Neither agree nor disagree | 7 | 18.9% | 10 | 34.5% |
|  | Agree | 15 | 40.5% | 9 | 31.0% |
|  | Strongly Agree | 7 | 18.9% | 0 | 0.0% |
|  | *Total* | *37* | *100.0%* | *29* | *100.0%* |
| Priorities in the health system draw attention away from HSPR research | Strongly Disagree | 1 | 2.7% | 3 | 10.0% |
|  | Disagree | 4 | 10.8% | 5 | 16.7% |
|  | Neither agree nor disagree | 10 | 27.0% | 9 | 30.0% |
|  | Agree | 14 | 37.8% | 9 | 30.0% |
|  | Strongly Agree | 8 | 21.6% | 4 | 13.3% |
|  | *Total* | *37* | *100.0%* | *30* | *100.0%* |
| Policymakers do not make decisions on the basis of HSPR research | Strongly Disagree | 1 | 2.7% | 3 | 10.3% |
|  | Disagree | 10 | 27.0% | 9 | 31.0% |
|  | Neither agree nor disagree | 7 | 18.9% | 10 | 34.5% |
|  | Agree | 16 | 43.2% | 6 | 20.7% |
|  | Strongly Agree | 3 | 8.1% | 1 | 3.4% |
|  | *Total* | *37* | *100.0%* | *29* | *100.0%* |
| Policymakers do not have technical access (i.e. journal subscriptions, links to research) to the appropriate databases to search for HSPR research | Strongly Disagree | 2 | 5.4% | 11 | 36.7% |
|  | Disagree | 7 | 18.9% | 12 | 40.0% |
|  | Neither agree nor disagree | 16 | 43.2% | 4 | 13.3% |
|  | Agree | 11 | 29.7% | 3 | 10.0% |
|  | Strongly Agree | 1 | 2.7% | 0 | 0.0% |
|  | *Total* | *37* | *100.0%* | *30* | *100.0%* |

**Table S3: Additional facilitators and barriers at the level of organizational support for KTE activities**

|  | | **Researchers** | | **Policy Makers** | |
| --- | --- | --- | --- | --- | --- |
|  |  | **N** | **%** | **N** | **%** |
| KT was hampered by a lack of incentives for KT activities within organization’s that conduct HSPR | Strongly Disagree | 1 | 2.7% | 2 | 7.7% |
|  | Disagree | 5 | 13.5% | 13 | 50.0% |
|  | Neither agree nor disagree | 17 | 45.9% | 7 | 26.9% |
|  | Agree | 9 | 24.3% | 4 | 15.4% |
|  | Strongly Agree | 5 | 13.5% | 0 | 0.0% |
|  | *Total* | *37* | *100.0%* | *26* | *100.0%* |
| Organizations that conduct HSPR made available financial and human resources to assist with KT activities | Strongly Disagree | 2 | 5.4% | 0 | 0.0% |
|  | Disagree | 12 | 32.4% | 4 | 16.7% |
|  | Neither agree nor disagree | 14 | 37.8% | 9 | 37.5% |
|  | Agree | 8 | 21.6% | 9 | 37.5% |
|  | Strongly Agree | 1 | 2.7% | 2 | 8.3% |
|  | *Total* | *37* | *100.0%* | *24* | *100.0%* |
| Organizations that conduct HSPR were not seen as a credible source of research | Strongly Disagree | 8 | 21.6% | 16 | 57.1% |
|  | Disagree | 17 | 45.9% | 7 | 25.0% |
|  | Neither agree nor disagree | 7 | 18.9% | 3 | 10.7% |
|  | Agree | 3 | 8.1% | 2 | 7.1% |
|  | Strongly Agree | 2 | 5.4% | 0 | 0.0% |
|  | *Total* | *37* | *100.0%* | *28* | *100.0%* |

**Table S4: Alignment of available research to needs of knowledge users**

|  | | **Researchers** | | **Policy Makers** | |
| --- | --- | --- | --- | --- | --- |
|  |  | **N** | **%** | **N** | **%** |
| Available research coincided with the needs and expectations of target audiences | Strongly Disagree | 0 | 0.0% | 1 | 3.7% |
|  | Disagree | 4 | 10.8% | 5 | 18.5% |
|  | Neither agree nor disagree | 14 | 37.8% | 11 | 40.7% |
|  | Agree | 16 | 43.2% | 8 | 29.6% |
|  | Strongly Agree | 3 | 8.1% | 2 | 7.4% |
|  | *Total* | *37* | *100.0%* | *27* | *100.0%* |
| Available research coincided with my country’s priorities (e.g., with a National Research Agenda) | Strongly Disagree | 0 | 0.0% | 2 | 7.4% |
|  | Disagree | 5 | 13.5% | 1 | 3.7% |
|  | Neither agree nor disagree | 16 | 43.2% | 11 | 40.7% |
|  | Agree | 13 | 35.1% | 12 | 44.4% |
|  | Strongly Agree | 3 | 8.1% | 1 | 3.7% |
|  | *Total* | *37* | *100.0%* | *27* | *100.0%* |
| Available research was not considered relevant by policymakers | Strongly Disagree | 3 | 8.3% | 6 | 22.2% |
|  | Disagree | 15 | 41.7% | 9 | 33.3% |
|  | Neither agree nor disagree | 8 | 22.2% | 9 | 33.3% |
|  | Agree | 9 | 25.0% | 3 | 11.1% |
|  | Strongly Agree | 1 | 2.8% | 0 | 0.0% |
|  | *Total* | *36* | *100.0%* | *27* | *100.0%* |
| Available research lacked credibility among target audiences | Strongly Disagree | 8 | 21.6% | 12 | 42.9% |
|  | Disagree | 13 | 35.1% | 9 | 32.1% |
|  | Neither agree nor disagree | 11 | 29.7% | 7 | 25.0% |
|  | Agree | 4 | 10.8% | 0 | 0.0% |
|  | Strongly Agree | 1 | 2.7% | 0 | 0.0% |
|  | *Total* | *37* | *100.0%* | *28* | *100.0%* |
| No research was ready for use | Strongly Disagree | 9 | 24.3% | 16 | 64.0% |
|  | Disagree | 9 | 24.3% | 5 | 20.0% |
|  | Neither agree nor disagree | 17 | 45.9% | 3 | 12.0% |
|  | Agree | 2 | 5.4% | 1 | 4.0% |
|  | Strongly Agree | 0 | 0.0% | 0 | 0.0% |
|  | *Total* | *37* | *100.0%* | *25* | *100.0%* |

**Table S5: Factors that influence health policymaking in Israel**

|  | | **Researchers** | | **Policy Makers** | |
| --- | --- | --- | --- | --- | --- |
|  |  | **N** | **%** | **N** | **%** |
| Broad challenges in government / provider relations hindered the health policymaking process. | Strongly Disagree | 1 | 2.8% | 1 | 3.1% |
|  | Disagree | 3 | 8.3% | 5 | 15.6% |
|  | Neither agree nor disagree | 7 | 19.4% | 7 | 21.9% |
|  | Agree | 20 | 55.6% | 9 | 28.1% |
|  | Strongly Agree | 5 | 13.9% | 10 | 31.3% |
|  | *Total* | *36* | *100.0%* | *32* | *100.0%* |
| Broad challenges in intergovernmental (i.e. Ministry of health, Ministry of Finance) relations hindered the health policymaking process. | Strongly Disagree | 0 | 0.0% | 1 | 3.1% |
|  | Disagree | 3 | 8.1% | 0 | 0.0% |
|  | Neither agree nor disagree | 6 | 16.2% | 2 | 6.3% |
|  | Agree | 17 | 45.9% | 13 | 40.6% |
|  | Strongly Agree | 11 | 29.7% | 16 | 50.0% |
|  | *Total* | *37* | *100.0%* | *32* | *100.0%* |
| Policy formulation is usually based on internal Ministry of Health discussions and ad hoc process rather than evidence based processes | Strongly Disagree | 0 | 0.0% | 2 | 6.3% |
|  | Disagree | 7 | 18.9% | 10 | 31.3% |
|  | Neither agree nor disagree | 7 | 18.9% | 9 | 28.1% |
|  | Agree | 18 | 48.6% | 11 | 34.4% |
|  | Strongly Agree | 5 | 13.5% | 0 | 0.0% |
|  | *Total* | *37* | *100.0%* | *32* | *100.0%* |

**Table S6: Groups or Factors that exert a strong influence on the health policymaking process**

|  | | **Researchers** | | **Policy Makers** | |
| --- | --- | --- | --- | --- | --- |
|  |  | **N** | **%** | **N** | **%** |
| Health insurance funds | Strongly Disagree | 0 | 0.0% | 0 | 0.0% |
|  | Disagree | 1 | 2.8% | 1 | 3.2% |
|  | Neither agree nor disagree | 2 | 5.6% | 6 | 19.4% |
|  | Agree | 13 | 36.1% | 13 | 41.9% |
|  | Strongly Agree | 20 | 55.6% | 11 | 35.5% |
|  | *Total* | *36* | *100.0%* | *31* | *100.0%* |
| Physician associations | Strongly Disagree | 0 | 0.0% | 1 | 3.1% |
|  | Disagree | 1 | 2.8% | 4 | 12.5% |
|  | Neither agree nor disagree | 3 | 8.3% | 8 | 25.0% |
|  | Agree | 26 | 72.2% | 16 | 50.0% |
|  | Strongly Agree | 6 | 16.7% | 3 | 9.4% |
|  | *Total* | *36* | *100.0%* | *32* | *100.0%* |
| Limited health funding (the economy) | Strongly Disagree | 0 | 0.0% | 0 | 0.0% |
|  | Disagree | 1 | 2.9% | 0 | 0.0% |
|  | Neither agree nor disagree | 3 | 8.8% | 0 | 0.0% |
|  | Agree | 16 | 47.1% | 9 | 28.1% |
|  | Strongly Agree | 14 | 41.2% | 23 | 71.9% |
|  | *Total* | *34* | *100.0%* | *32* | *100.0%* |
| Media | Strongly Disagree | 0 | 0.0% | 0 | 0.0% |
|  | Disagree | 2 | 5.6% | 1 | 3.2% |
|  | Neither agree nor disagree | 9 | 25.0% | 8 | 25.8% |
|  | Agree | 20 | 55.6% | 17 | 54.8% |
|  | Strongly Agree | 5 | 13.9% | 5 | 16.1% |
|  | *Total* | *36* | *100.0%* | *31* | *100.0%* |
| Values of governing parties | Strongly Disagree | 0 | 0.0% | 7 | 21.9% |
|  | Disagree | 3 | 8.3% | 4 | 12.5% |
|  | Neither agree nor disagree | 11 | 30.6% | 8 | 25.0% |
|  | Agree | 12 | 33.3% | 9 | 28.1% |
|  | Strongly Agree | 10 | 27.8% | 4 | 12.5% |
|  | *Total* | *36* | *100.0%* | *32* | *100.0%* |
| Public opinion | Strongly Disagree | 1 | 2.8% | 1 | 3.1% |
|  | Disagree | 5 | 13.9% | 6 | 18.8% |
|  | Neither agree nor disagree | 11 | 30.6% | 13 | 40.6% |
|  | Agree | 18 | 50.0% | 11 | 34.4% |
|  | Strongly Agree | 1 | 2.8% | 1 | 3.1% |
|  | *Total* | *36* | *100.0%* | *32* | *100.0%* |
| Nursing associations | Strongly Disagree | 2 | 5.7% | 1 | 3.1% |
|  | Disagree | 6 | 17.1% | 13 | 40.6% |
|  | Neither agree nor disagree | 11 | 31.4% | 8 | 25.0% |
|  | Agree | 11 | 31.4% | 6 | 18.8% |
|  | Strongly Agree | 5 | 14.3% | 4 | 12.5% |
|  | *Total* | *35* | *100.0%* | *32* | *100.0%* |
| Research about problems related to healthcare or health systems | Strongly Disagree | 1 | 2.8% | 1 | 3.2% |
|  | Disagree | 8 | 22.2% | 7 | 22.6% |
|  | Neither agree nor disagree | 13 | 36.1% | 17 | 54.8% |
|  | Agree | 14 | 38.9% | 6 | 19.4% |
|  | Strongly Agree | 0 | 0.0% | 0 | 0.0% |
|  | *Total* | *36* | *100.0%* | *31* | *100.0%* |
| Other countries’ health policies | Strongly Disagree | 0 | 0.0% | 0 | 0.0% |
|  | Disagree | 7 | 19.4% | 5 | 16.1% |
|  | Neither agree nor disagree | 18 | 50.0% | 15 | 48.4% |
|  | Agree | 10 | 27.8% | 8 | 25.8% |
|  | Strongly Agree | 1 | 2.8% | 3 | 9.7% |
|  | *Total* | *36* | *100.0%* | *31* | *100.0%* |
| Donor organizations | Strongly Disagree | 1 | 2.8% | 4 | 12.5% |
|  | Disagree | 14 | 38.9% | 13 | 40.6% |
|  | Neither agree nor disagree | 13 | 36.1% | 14 | 43.8% |
|  | Agree | 6 | 16.7% | 1 | 3.1% |
|  | Strongly Agree | 2 | 5.6% | 0 | 0.0% |
|  | *Total* | *36* | *100.0%* | *32* | *100.0%* |
| Other types of health professional associations | Strongly Disagree | 2 | 5.6% | 5 | 16.7% |
|  | Disagree | 7 | 19.4% | 12 | 40.0% |
|  | Neither agree nor disagree | 19 | 52.8% | 7 | 23.3% |
|  | Agree | 8 | 22.2% | 5 | 16.7% |
|  | Strongly Agree | 0 | 0.0% | 1 | 3.3% |
|  | *Total* | *36* | *100.0%* | *30* | *100.0%* |

**Table S7: Engagement with KTE activities**

| **Researchers** | | | | **Policy Makers** | | | |
| --- | --- | --- | --- | --- | --- | --- | --- |
|  | | **N** | **%** | **N** | **%** |  | |
| Provided copies of articles and/or systematic reviews about HSPR to policymakers and/or decision makers | never | 3 | 8.6% | 0 | 0.0% | never | Received copies of articles and/or systematic reviews about HSPR to policymakers and/or decision makers |
|  | rarely | 10 | 28.6% | 4 | 12.9% | rarely |  |
|  | occasionally | 13 | 37.1% | 7 | 22.6% | occasionally |  |
|  | frequently | 8 | 22.9% | 12 | 38.7% | frequently |  |
|  | always | 1 | 2.9% | 8 | 25.8% | always |  |
|  | *Total* | *35* | *100.0%* | *31* | *100.0%* | *Total* |  |
| Provided mailings or e-mails that the content was tailored to specific policymakers and/or decision makers | never | 7 | 20.0% | 2 | 6.5% | never | Received mailings or e-mails that the content was tailored to specific policymakers and/or decision makers |
|  | rarely | 11 | 31.4% | 6 | 19.4% | rarely |  |
|  | occasionally | 8 | 22.9% | 6 | 19.4% | occasionally |  |
|  | frequently | 8 | 22.9% | 14 | 45.2% | frequently |  |
|  | always | 1 | 2.9% | 3 | 9.7% | always |  |
|  | *Total* | *35* | *100.0%* | *31* | *100.0%* | *Total* |  |
| Interacted with credible messengers/sources outside your organization to promote HSPR | never | 6 | 17.6% | 0 | 0.0% | never | Interacted with credible messengers/sources outside your organization to obtain HSPR |
|  | rarely | 8 | 23.5% | 1 | 3.2% | rarely |  |
|  | occasionally | 10 | 29.4% | 9 | 29.0% | occasionally |  |
|  | frequently | 9 | 26.5% | 13 | 41.9% | frequently |  |
|  | always | 1 | 2.9% | 8 | 25.8% | always |  |
|  | *Total* | *34* | *100.0%* | *31* | *100.0%* | *Total* |  |
| Provided articles, reports, syntheses, formal systematic reviews and/or messages to policymakers and/or decision makers without an explicit request | never | 3 | 8.8% | 2 | 6.5% | never | Received articles, reports, syntheses, formal systematic reviews and/or messages to policymakers and/or decision makers without an explicit request |
|  | rarely | 11 | 32.4% | 8 | 25.8% | rarely |  |
|  | occasionally | 13 | 38.2% | 8 | 25.8% | occasionally |  |
|  | frequently | 6 | 17.6% | 9 | 29.0% | frequently |  |
|  | always | 1 | 2.9% | 4 | 12.9% | always |  |
|  | *Total* | *34* | *100.0%* | *31* | *100.0%* | *Total* |  |
| Interacted with policymakers and/or decision makers when developing a specific research question, objectives or hypothesis | never | 2 | 5.9% | 0 | 0.0% | never | Interacted with researchers when developing a specific research question, objectives or hypothesis |
|  | rarely | 14 | 41.2% | 6 | 19.4% | rarely |  |
|  | occasionally | 6 | 17.6% | 9 | 29.0% | occasionally |  |
|  | frequently | 12 | 35.3% | 11 | 35.5% | frequently |  |
|  | always | 0 | 0.0% | 5 | 16.1% | always |  |
|  | *Total* | *34* | *100.0%* | *31* | *100.0%* | *Total* |  |
| Interacted with policymakers and/or decision makers through events organized by them or their organization or though informal conversations | never | 1 | 2.9% | 0 | 0.0% | never | Interacted with researchers through events organized by them or their organization or though informal conversations |
|  | rarely | 6 | 17.6% | 3 | 9.7% | rarely |  |
|  | occasionally | 14 | 41.2% | 6 | 19.4% | occasionally |  |
|  | frequently | 10 | 29.4% | 13 | 41.9% | frequently |  |
|  | always | 3 | 8.8% | 9 | 29.0% | always |  |
|  | *Total* | *34* | *100.0%* | *31* | *100.0%* | *Total* |  |
| Assessed or participated in assessments of the usefulness and impact of your KTE activities | never | 10 | 29.4% | 5 | 16.7% | never | Assessed or participated in assessments of the usefulness and impact of your KTE activities |
|  | rarely | 13 | 38.2% | 5 | 16.7% | rarely |  |
|  | occasionally | 7 | 20.6% | 6 | 20.0% | occasionally |  |
|  | frequently | 4 | 11.8% | 8 | 26.7% | frequently |  |
|  | always | 0 | 0.0% | 6 | 20.0% | always |  |
|  | *Total* | *34* | *100.0%* | *30* | *100.0%* | *Total* |  |
| Provided access to a searchable database of articles, reports, syntheses, and or formal systematic reviews on HSPR | never | 13 | 38.2% | 2 | 6.5% | never | Received/ had access to a searchable database of articles, reports, syntheses, and or formal systematic reviews on HSPR |
|  | rarely | 14 | 41.2% | 2 | 6.5% | rarely |  |
|  | occasionally | 4 | 11.8% | 3 | 9.7% | occasionally |  |
|  | frequently | 3 | 8.8% | 9 | 29.0% | frequently |  |
|  | always | 0 | 0.0% | 15 | 48.4% | always |  |
|  | *Total* | *34* | *100.0%* | *31* | *100.0%* | *Total* |  |
| Provided training to policymakers and/or decision makers to develop their capacity to acquire, assess, adapt and apply HSPR. | never | 12 | 35.3% | 10 | 33.3% | never | Received training to policymakers and/or decision makers to develop their capacity to acquire, assess, adapt and apply HSPR. |
|  | rarely | 12 | 35.3% | 6 | 20.0% | rarely |  |
|  | occasionally | 6 | 17.6% | 6 | 20.0% | occasionally |  |
|  | frequently | 4 | 11.8% | 5 | 16.7% | frequently |  |
|  | always | 0 | 0.0% | 3 | 10.0% | always |  |
|  | *Total* | *34* | *100.0%* | *30* | *100.0%* | *Total* |  |
| Established and/or maintained long term partnerships with HSPR policymakers and/or decision makers (e.g., through an advisory board) | never | 5 | 14.7% | 0 | 0.0% | never | Established and/or maintained long term partnerships with HSPR researchers (e.g., through an advisory board) |
|  | rarely | 9 | 26.5% | 6 | 20.0% | rarely |  |
|  | occasionally | 10 | 29.4% | 4 | 13.3% | occasionally |  |
|  | frequently | 9 | 26.5% | 10 | 33.3% | frequently |  |
|  | always | 1 | 2.9% | 10 | 33.3% | always |  |
|  | *Total* | *34* | *100.0%* | *30* | *100.0%* | *Total* |  |
